# Forearm Supination Increases the Safety Corridor and Target Exposure for Ultrasound-Guided Flexor Pollicis Longus Injection: A Prospective Study in Healthy Adults

**DOI:** 10.3390/diagnostics16162674

**Published:** 2026-08-21

**Authors:** Hoon Ki Song, Hyo Jung Kang, Woo-Hwa Choi, Ho-Yong Jeong, Chi Hwan An, Hyokyum Kim

**Affiliations:** 1 Department of Physical Medicine and Rehabilitation, Veterans Health Service Medical Center, Seoul 05368, Republic of Korea; thdgnsrl5018@naver.com (H.K.S.); amander2@naver.com (W.-H.C.); ghdyd8623@gmail.com (H.-Y.J.); espoir3054@gmail.com (C.H.A.); miniking00@naver.com (H.K.); 2 Department of Medicine, Graduate School, Korea University, Seoul 02841, Republic of Korea

**Keywords:** spasticity, botulinum toxin, flexor pollicis longus, ultrasound-guided injection, forearm supination, safety corridor

## Abstract

**Background**/**Objectives**: Ultrasound-guided flexor pollicis longus (FPL) injection is limited by the muscle’s deep location and its proximity to the cephalic vein (CV) and superficial branch of the radial nerve (SBRN). We aimed to identify the wrist and forearm posture producing the largest measured corridor width and target exposure. **Methods**: This prospective single-center study evaluated 51 forearms from 27 healthy adult volunteers with full range of motion, using transverse ultrasound of the distal volar forearm in six standardized positions. Two corridors were measured: CV-to-SBRN (L_1_, Approach A) and CV-to-radial-cortex apex (L_2_, Approach B), with FPL exposure height (F). **Results**: L_2_ was present in all limbs and was wider with forearm supination than in neutral (supination [SUP], +2.36 mm; flexion plus supination [F+SUP], +2.05 mm; extension plus supination [E+SUP], +2.66 mm; each *p* < 0.001), an ~40% increase; the three supination postures did not differ. F increased across the same postures and was greatest in E+SUP (+2.32 mm), which did not differ from SUP. L_1_ was absent in 12–28% of limbs and, where present, was narrower with supination (both *p* < 0.001). **Conclusions**: In healthy adults, forearm supination widened the safety corridor and increased target exposure. Applicability to spasticity requires confirmation.

## 1. Introduction

Spasticity, defined as a velocity-dependent increase in tonic stretch reflexes resulting from upper motor neuron lesions, is a prevalent and disabling sequela of neurological disorders, including stroke, cerebral palsy, and spinal cord injury [1,2]. Following stroke, upper limb spasticity affects 17–39% of survivors and can produce characteristic deformity patterns, functional impairment, pain, and impaired hygiene [3,4]. Among the upper extremity muscles implicated in post-stroke spasticity, the flexor pollicis longus (FPL) is critical in thumb flexion. FPL hypertonicity manifests as a thumb-in-palm deformity, impairing prehensile function, promoting palmar skin maceration, and, in severe cases, skin breakdown [5,6].

Botulinum toxin type A (BoNT-A) has emerged as a highly effective treatment for managing focal spasticity, reducing muscle tone with a favorable safety profile and reversible effects [7,8,9]. Whereas oral antispastic agents such as baclofen and tizanidine provide systemic coverage, their use is frequently limited by sedation and cognitive side effects; conversely, focal injection therapy circumvents these limitations by delivering targeted chemodenervation [10]. Moreover, advances in high-resolution musculoskeletal ultrasonography have enabled real-time visualization of target muscles and adjacent neurovascular structures during injection, and ultrasound-guided BoNT-A injection has increasingly become the preferred approach in practice [11,12,13]. Electromyography (EMG)- and electrical stimulation-guided techniques nonetheless remain established methods and continue to be used, either alone or together with ultrasound, and the relative merits of the available guidance techniques remain a subject of ongoing comparison [11]. As such, the property of ultrasound that is relevant to the present study is not accuracy of muscle localization, but the fact that it displays the neurovascular structures lying along the intended needle path.

The FPL originates from the anterior surface of the radius and the adjacent interosseous membrane, traversing the carpal tunnel to insert at the base of the distal phalanx of the thumb [14]. In the distal forearm, it lies deep, immediately medial to the radial cortex, producing a distinctive “shark fin” appearance on transverse ultrasound imaging [15]. In patients with upper-limb spasticity, the forearm is often held in a pronated position, which limits ulnar-side access and makes the radial side the more practical injection route [16]. Nonetheless, this anatomical configuration poses particular challenges for radial-side injection access. Specifically, three closely adjacent structures require avoidance: the cephalic vein (CV) and the superficial branch of the radial nerve (SBRN), which traverse the radial aspect of the distal forearm in intimate apposition [17,18,19], and the radial cortex, which may physically obstruct needle advancement toward the FPL muscle belly. Because the structures bordering the FPL at this level lie within less than 1 cm of one another, the windows available for needle passage are correspondingly narrow, and accurate guidance is required if puncture of the adjacent vessel or nerve—with the attendant risks of local hematoma formation and nerve injury—is to be avoided [15].

Beyond therapeutic injection, the FPL is a diagnostically important muscle sampled by needle EMG in anterior interosseous neuropathy [20]. Standardized approaches to the FPL have relied on landmark-based, perpendicular needle insertion at the junction of the middle and distal thirds of the radial forearm [20,21]. Because the needle is advanced without real-time imaging and the CV, SBRN, and radial artery lie close to the muscle at this level, avoidance of these structures depends entirely on individual anatomy and cannot be confirmed; indeed, surface-landmark localization of the FPL has been shown to place the injection target significantly more proximally than the ultrasound-determined optimal site [22].

The in-plane ultrasound-guided technique addresses these shortcomings by advancing the needle parallel to the transducer, so the entire needle shaft and tip, together with the target, remain continuously visualized, allowing real-time avoidance of the CV, SBRN, and radial cortex regardless of anatomical variation [23]. Nevertheless, maintaining coaxial needle-beam alignment in-plane is technically demanding, and within the narrow confines of the distal radial forearm, the out-of-plane approach has often been preferred for FPL injection [13,15]. The difficulty specific to the FPL is that the muscle lies partly beneath the radial cortex amid closely apposed neurovascular structures, creating a confined channel through which the needle must pass. Throughout this study we refer to this channel as the safety corridor. We use the term in a strictly sonographic sense: it denotes the width, measured on a single transverse ultrasound plane, of the space separating the structures that must be avoided. It is therefore an indirect anatomical surrogate of accessibility rather than a validated measure of procedural safety. Because the structures bordering this corridor shift relative to one another with changes in limb posture, positioning provides a direct, modifiable means of widening it; a wider corridor would, in turn, reduce the spatial constraint on needle angulation and allow the continuous full-needle visualization of the in-plane approach to be used safely.

Existing ultrasonographic descriptions of the FPL have improved target localization—whether for diagnostic EMG [21] or therapeutic injection [22], yet they characterize the relevant anatomy in a single, neutral posture. Consequently, the dynamic repositioning of the FPL and its neighboring structures during wrist and forearm motion remains uncharacterized.

That such repositioning is likely to be substantial is indicated by work on neighboring structures. Using high-resolution ultrasound in 55 asymptomatic participants, Mohana-Borges et al. showed that the extremes of forearm rotation alter the longitudinal alignment and anatomical relationships of the deep branch of the radial nerve, with a mean angular difference of approximately 7° between maximal supination and maximal pronation [24]. That study concerned a different branch of the radial nerve at a more proximal level, and its measurements cannot be transferred to the distal forearm; it does, however, establish that forearm rotation is not neutral with respect to the sonographic anatomy of the radial nerve. For the two structures of direct relevance here, Kim et al. characterized the course of the SBRN and its relationship to the CV in the distal forearm, but examined the limb with the forearm held midway between pronation and supination; they noted that their measurements might change with forearm position and identified this as a question requiring further study [17].

In other ultrasound-guided procedures, joint motion has been used to enlarge the safety corridor; during stellate ganglion block, for example, contralateral neck rotation significantly increases the distance between the procedural target and the common carotid artery, thereby reducing the risk of inadvertent arterial puncture [25]. Such a dynamic, motion-based strategy has not been applied to ultrasound-guided FPL injection, which has so far relied on a single neutral wrist position. A more dynamic use of adjacent joint motion to actively widen the FPL safety corridor is therefore needed.

This study does not propose a new injection technique. The ultrasound-guided approach, the target muscle, the equipment, and the needle trajectory are all unchanged. What is examined here is whether a variable that is already under the operator’s control—the position of the wrist and forearm at the moment of injection—can be selected so as to widen the space within which the existing technique must be performed.

The study was accordingly organized around a defined hierarchy of questions. Across six standardized wrist and forearm positions, we quantified two candidate corridors on transverse ultrasound: the space between the CV and the SBRN (L_1_, Approach A), and the space between the CV and the apex of the radial cortex (L_2_, Approach B). We also measured the exposed height of the FPL above the radial cortex (F). L_2_ and F were treated as co-primary outcomes because they represent two aspects of a single question: whether the needle can reach the muscle, and whether a sufficient extent of muscle is available once it has been reached. L_1_ was a further confirmatory outcome and was analyzed separately, since the corridor it describes proved to be absent in a proportion of limbs. Analyses involving participant characteristics—age, sex, and body mass index—were exploratory throughout and are not used to support any conclusion. By identifying the posture associated with the largest sonographic dimensions among those examined, we sought to provide preliminary anatomical evidence to inform safer ultrasound-guided FPL injection.

## 2. Materials and Methods

### 2.1. Study Design and Ethics

This prospective, single-center observational study was conducted in accordance with the Declaration of Helsinki. Ethical approval was obtained from the Institutional Review Board of Veterans Health Service Medical Center (Seoul, Republic of Korea; IRB No. 2023-11-008-002), and written informed consent was obtained from all participants before enrollment.

### 2.2. Participants

Between July 2025 and December 2025, participants were consecutively recruited based on the following inclusion criteria: (1) adults aged 18 years or older with no limitations in activities of daily living; (2) patients undergoing musculoskeletal ultrasound of other body parts; and (3) individuals who fully understood the study purpose and voluntarily provided written informed consent. The exclusion criteria were (1) a history of musculoskeletal disorders causing structural deformities of the upper extremity, such as fractures or joint surgeries; (2) concomitant neurologic abnormalities, including muscle weakness or sensory changes; and (3) refusal to undergo additional ultrasound examinations for the study. Initially, 29 participants (17 males and 12 females) were recruited. During the initial screening, performed with the wrist and forearm in the neutral position before the positional sequence began, two participants (2 males) were excluded because the SBRN could not be distinguished from the surrounding connective tissue and its course could not be traced anywhere along the forearm; in both, this was observed bilaterally, suggesting a characteristic of the individual rather than a limb-specific finding. Among the remaining 27 participants (54 limbs), 3 further limbs were excluded at the stage of establishing the target scanning level. With the limb in the neutral position, the target level was identified as the level at which the FPL was maximally exposed above the radial cortex; a limb proceeded to the positional sequence only if both the SBRN and the CV were simultaneously visible within the field of view at that level. In contrast to the two participants excluded at screening, the nerve could be traced along the forearm in these limbs, but one or other landmark could not be localized within the field of view at the target level itself—the SBRN in one limb and the CV in two, the latter reflecting venous variation. Because this determination was made before the positional sequence began, no images were acquired in these limbs, and exclusion applied to the limb as a whole rather than to individual positions. Every included limb therefore contributed complete measurements in all six positions, with no missing values. Consequently, a final total of 51 limbs from 27 participants was included in the analysis.

Both limbs were examined in participants who consented to bilateral assessment; 24 of the 27 participants contributed two limbs and 3 contributed one. Examining both limbs increases the number of observations available for estimating positional effects without additional recruitment. The resulting within-participant correlation was not ignored but modeled directly, by including a random intercept for limb nested within participant (Section 2.7.1), so that the effective sample size for participant-level characteristics remains 27.

Healthy adults rather than patients with spasticity were studied by design. Spasticity alters muscle volume and architecture, and the degree of change varies considerably between individuals; measurements obtained in such a population could not be interpreted without a normative reference, since it would not be possible to distinguish variation attributable to the condition from variation attributable to individual anatomy. We therefore sought first to establish how the relevant structures are related in the unaffected forearm and how those relationships change with wrist and forearm position, so that subsequent work in patients with spasticity has a baseline against which to be compared.

### 2.3. Ultrasound Examination Protocol

All ultrasound examinations were performed by a single experienced musculoskeletal sonographer with over 10 years of musculoskeletal ultrasound experience to ensure procedural consistency, using a high-resolution linear transducer (5–12 MHz; Accuvix XQ, Medison Co., Ltd., Seoul, Republic of Korea). To minimize transducer-induced compression artifacts, a generous amount of acoustic coupling gel was applied at each measurement site, thereby preserving near-field tissue geometry and avoiding artificial deformation of superficial structures [26]. Gain, compression, and time-gain compensation settings were kept constant throughout the study. Scanning depth was standardized at 3.0 cm to optimize visualization. Transducer pressure was not quantified. The examiner applied the minimum pressure at which acoustic contact was maintained and confirmed on the screen at each acquisition that the outline of the CV was not deformed by compression; this was a visual judgment rather than a measured criterion. Because the transducer was lifted and replaced at the marked level before each position (Section 2.4), pressure was not applied cumulatively across the sequence.

Measurements were obtained with the transducer positioned in the transverse plane over the radial aspect of the distal third of the volar forearm. The scanning level was determined once, at the start of the examination with the limb in the neutral position, and was defined as the level at which the FPL was maximally exposed above the radial cortex, appearing as a discrete muscle mass medial to the radial cortex. This level was adopted because it is the site at which the FPL is injected in our clinical practice; it corresponds to the injection site described in published ultrasound guides for this muscle, in which the toxin is delivered at the point of maximum muscle thickness in the radial portion of the distal third of the forearm [15]. Because the examination required the limb to be moved through six positions, this level was marked on the skin at the neutral position, and all six acquisitions were obtained at the same marked level. The scanning level was therefore not re-identified after each positional change: a single longitudinal level of the forearm was fixed throughout, so that the six acquisitions differed only in wrist and forearm position. This design was intentional, since the aim was to determine how a change in posture alters the spatial relationships among the landmark structures at one and the same anatomical level.

The transducer was applied perpendicular to the skin surface at each measurement site, rather than tilted to align the beam with any single structure, so that the spatial relationships among all structures were captured within one image. The radial cortex, used as the reference landmark for L_2_, is a strong specular reflector whose brightness falls off sharply once the beam deviates from perpendicular incidence; maximal cortical brightness on the screen therefore served as the examiner’s indicator of correct transducer orientation at each acquisition.

Before any image was stored, the transducer was moved proximally and distally along the forearm so that the CV and the SBRN could be followed along their courses and identified by continuity rather than from a single static section. This step was necessary for the SBRN in particular: unlike a vessel it has neither a lumen nor pulsation by which it can be recognized, and identification from one static image is unreliable. Dynamic image interpretation has been shown to improve the sonographic differentiation of nerve structures relative to static reading [27]. Only once both structures had been identified in this way was the transducer returned to the marked level and the image acquired. The limbs excluded at this stage, in which one or other structure could not be localized at the target level, were identified by this procedure (Section 2.2).

### 2.4. Wrist and Forearm Positioning Protocol

Each participant underwent sequential ultrasound assessment across six standardized wrist and forearm positions, performed in a fixed order to minimize fatigue-related variability:

1. Neutral (NS): forearm and wrist in neutral position;
2. Supination (SUP): maximal forearm supination;
3. Flexion (F*): maximal wrist flexion in neutral forearm rotation;
4. Extension (E): maximal wrist extension in neutral forearm rotation;
5. Flexion with supination (F+SUP): maximal wrist flexion combined with maximal forearm supination;
6. Extension with supination (E+SUP): maximal wrist extension combined with maximal forearm supination.

Throughout the manuscript, wrist flexion is denoted F* to distinguish it from F, the FPL exposure height. Each position was taken to the participant’s maximal available range rather than to a fixed angular target, and joint angles were not measured. Participants were examined in the supine position, with the forearm resting on the examination table so that the limb was comfortably supported and stable throughout the examination; the limb was not held or elevated by the examiner. Before each position was assumed, the limb was returned to the neutral position so that the transducer could be replaced perpendicular to the skin at the marked scanning level; the position was then adopted and the image acquired immediately, without an intervening rest interval. Each of the six positions was therefore approached from the same neutral starting point rather than sequentially from the preceding position. Each position was passively maintained by the examiner during image acquisition. Because all positions were assumed passively, with the examiner moving the limb through the range while the participant remained inactive, the forearm musculature was relaxed throughout image acquisition. Participants were instructed not to assist the movement or to hold the position actively.

### 2.5. Outcome Measures

**Figure 1:**
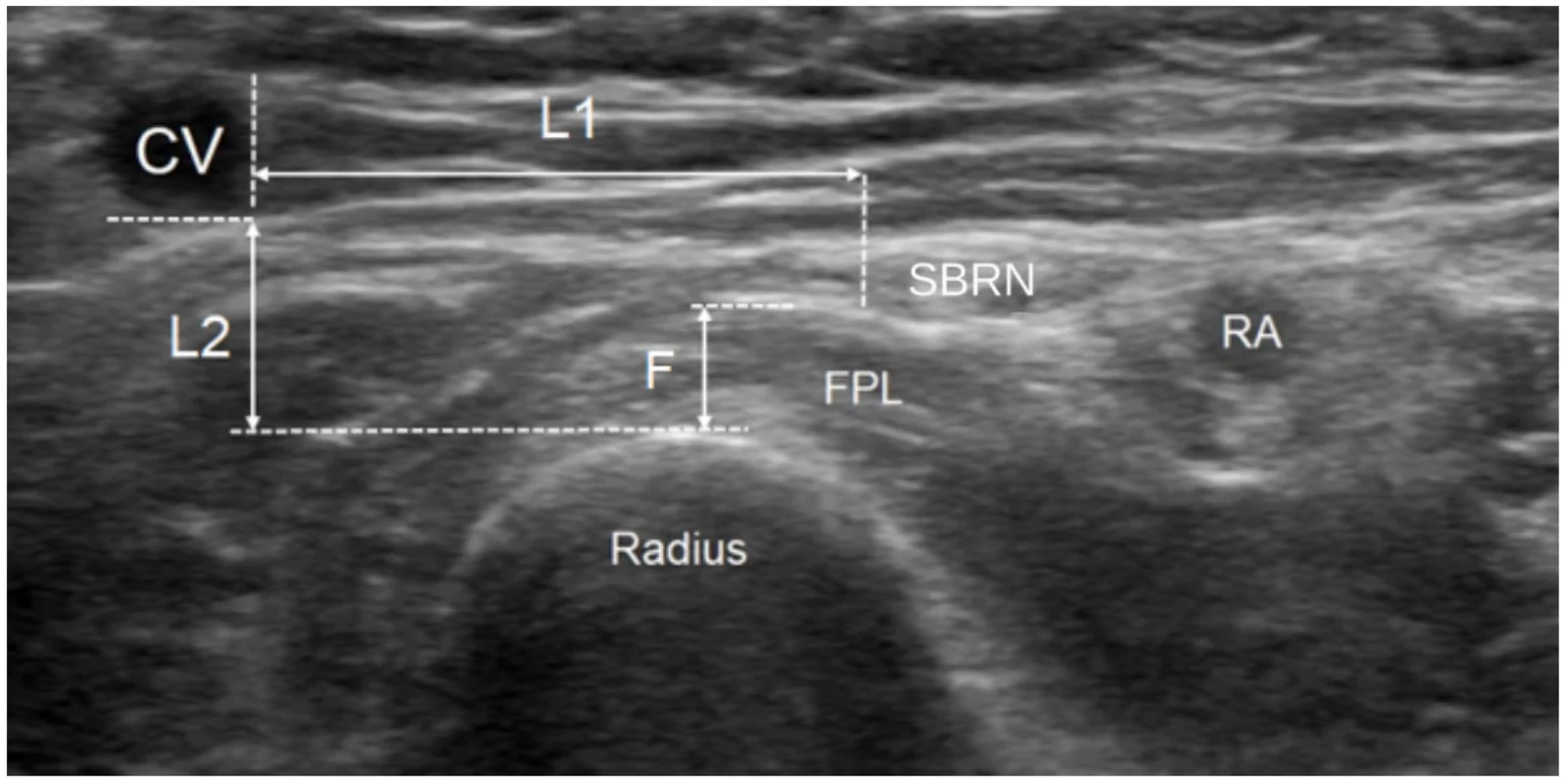
Transverse ultrasonographic view of the distal third of the forearm. Solid double-headed arrows indicate the measured distances; dashed lines mark the reference borders from which each measurement was taken. L_1_, distance between the medial wall of the CV and the lateral border of the SBRN; L_2_, distance between the inferior wall of the CV and the apex (i.e., the highest visible point) of the radial cortex; F, FPL exposure height. CV, cephalic vein; FPL, flexor pollicis longus; RA, radial artery; SBRN, superficial branch of the radial nerve.

**Figure 2:**
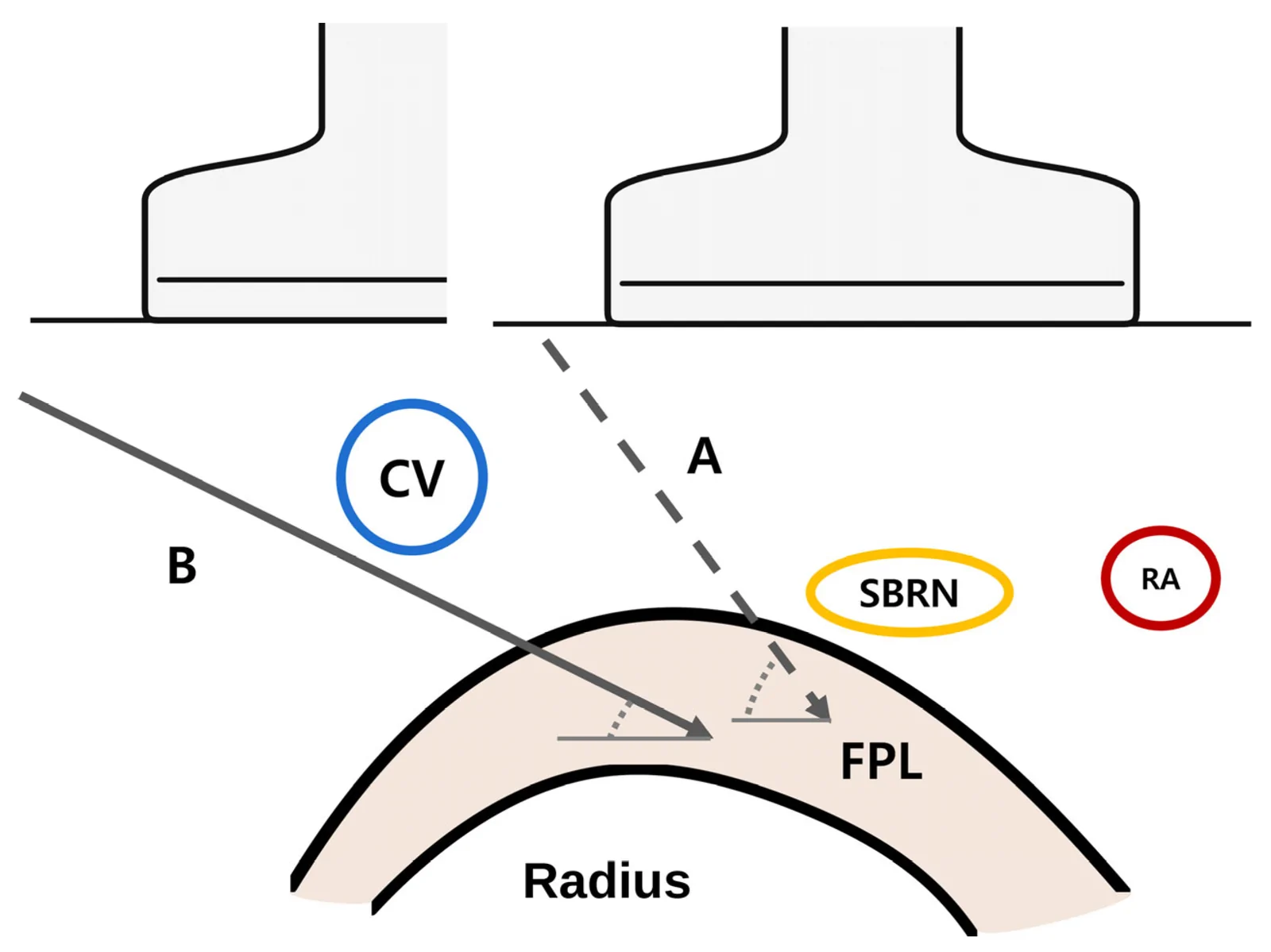
Schematic representation of the two ultrasound-guided injection approaches to the flexor pollicis longus in the distal forearm, shown in the transverse plane with the transducer applied perpendicular to the skin. In both approaches the needle is advanced in-plane, within the plane of the ultrasound beam, so that the shaft and tip remain continuously visualized. Approach A (A) passes between the cephalic vein and the superficial branch of the radial nerve (corridor L_1_); Approach B (B) passes beneath the cephalic vein and above the apex of the radial cortex (corridor L_2_). The needle entry angle is steeper for Approach A than for Approach B. The radial artery lies medial to the target and is not crossed by either trajectory. A, Approach A; B, Approach B; CV, cephalic vein; FPL, flexor pollicis longus; RA, radial artery; SBRN, superficial branch of the radial nerve.

The primary ultrasound outcome measures were assessed for each of the six wrist and forearm positions and for both injection approaches (Figure 1); the two approaches are shown schematically in Figure 2. Three measurements were made. Two of them describe whether the needle can be brought to the muscle at all—L_1_ for Approach A and L_2_ for Approach B—and the third describes how much muscle is available once it has been reached (F). Quantities that depend on where the operator elects to enter the skin, such as the angle of insertion or the length of the intramuscular path, were not measured: they are properties of a particular insertion rather than of the imaged anatomy, and at any given posture they would vary with operator preference. Both L_1_ and L_2_ are cross-sectional surrogates of accessibility, as defined in the Introduction section. All measurements were performed on stored transverse images using ImageJ version 1.54g (National Institutes of Health, Bethesda, MD, USA) [28].

L_1_ (Approach A corridor): the horizontal distance, in mm, between the medial wall of the CV and the lateral border of the SBRN, measured on transverse ultrasound imaging. L_1_ was used as a cross-sectional surrogate of the space available for needle passage between these two structures. At the target scanning level in the distal third of the volar forearm, the SBRN appeared as a single, discrete hyperechoic fascicular structure in all included limbs, and the lateral border was taken as the lateral margin of that structure; division into separately identifiable branches was not observed at this level in this cohort. L_1_ was recorded as zero when both structures were identifiable but no usable corridor was present between them: when the two structures were in direct apposition with no intervening space, and when their mediolateral order was reversed relative to the configuration shown in Figure 1. A zero value therefore denotes the operational absence of a usable corridor for Approach A rather than a literal 0 mm separation. This coding was adopted because Approach A depends on a fixed spatial relationship between the two landmarks; where that relationship is absent or inverted, the landmark configuration on which the approach is based does not hold. Limbs in which either structure could not be identified within the field of view at the target scanning level were excluded rather than coded as zero (Section 2.2), since in those limbs the corridor could not be assessed at all.

L_2_ (Approach B corridor): the horizontal distance between the inferior wall of the CV and the apex (highest visible point) of the radial cortex on transverse ultrasound imaging. L_2_ was used as a cross-sectional surrogate of the space available for Approach B, in which the needle is routed between the CV and the bony radial surface to avoid the SBRN.

F (FPL exposure height): F was defined as the sonographic height of the muscle visible above the radial cortex on transverse imaging [29]. Greater F values indicate that a larger portion of the muscle is visible above the radial cortex on the imaged plane. Whether this corresponds to a larger injectable cross-sectional area or to greater technical ease of needle insertion was not assessed in this study.

Baseline participant characteristics, including age, sex, height, weight, and body mass index (BMI), were recorded.

### 2.6. Reliability Assessment

All ultrasound images were acquired by a single sonographer with over 10 years of musculoskeletal ultrasound experience. Each acquired image was then independently measured by two raters (with over 10 and over 2 years of experience, respectively), each of whom repeated every measurement three times. The mean of the three repetitions was taken as each rater’s value, and the mean of the two raters’ values was used as the final measurement for analysis. Measurement reproducibility was quantified using intraclass correlation coefficients (ICCs) with 95% confidence intervals under a two-way mixed-effects, absolute-agreement model. Intra-rater reliability was assessed from the three repeated measurements of each rater (ICC(A,1)). Inter-rater reliability was assessed from the two raters’ mean values and is reported primarily as an average-measures estimate (ICC(A,2)), which corresponds to the two-rater mean used in the analysis, with the single-measures estimate (ICC(A,1)) also provided. ICCs were interpreted according to Koo and Li [30] as poor (<0.50), moderate (0.50–0.75), good (0.75–0.90), or excellent (>0.90).

Because all images were acquired by a single sonographer and both raters measured the same acquired images, these coefficients quantify the repeatability of measurement on stored images. They do not characterize the reproducibility of image acquisition or the stability of the anatomical imaging plane, which would require independent rescanning by more than one operator and was not undertaken in this study.

### 2.7. Statistical Analysis

Analyses were performed using SPSS 26.0 (IBM Corp., Armonk, NY, USA) and Python 3.12 (Python Software Foundation, Wilmington, DE, USA) with SciPy 1.11, Pingouin 0.5, and Statsmodels 0.14. The mixed models and generalized estimating equations were fitted in Python with Statsmodels 0.14. Continuous variables are presented as mean ± SD. All tests were two-sided, with significance set at *p* < 0.05.

#### 2.7.1. Data Structure and Model Specification

The observational unit was the limb, and each limb was imaged in six positions. The data therefore had a three-level hierarchy: repeated observations nested within limbs, and limbs nested within participants. The analyses of L_2_, F, and of the conditional corridor width in Part 2 of the L_1_ analysis (Section 2.7.2) used mixed-effects models with a random intercept for participant and a random intercept for limb nested within participant, with a compound-symmetric covariance structure assumed at each level. The adequacy of this structure was confirmed by likelihood ratio tests against a model with a participant-level random intercept only (L_2_, χ^2^ = 93.7; F, χ^2^ = 19.1; both *p* < 0.001). L_2_ and F were analyzed with position as a six-level fixed effect (neutral as reference). Final parameter estimates were obtained by restricted maximum likelihood (REML); models differing in fixed effects were re-estimated by full maximum likelihood for likelihood ratio testing, as REML likelihoods are not comparable across different fixed-effect structures.

#### 2.7.2. Analysis of L_1_

L_1_ was analyzed with a two-part (hurdle) approach because the corridor was either anatomically absent (L_1_ = 0) or present with a measurable width, and the subset of limbs contributing to the width analysis differed across positions. Part one modeled the probability that the corridor was absent (L_1_ = 0) by logistic regression estimated with generalized estimating equations (GEE), specifying position as a six-level fixed effect (neutral as the reference), the limb as the clustering unit, an exchangeable working correlation for the six repeated binary observations within a limb, and robust (sandwich) standard errors. This part contained no random effects; its coefficients are therefore population-averaged log-odds, and within-limb dependence is accommodated through the working correlation and the robust variance estimator. The overall effect of position was assessed by a multivariable Wald test of the five position coefficients, and a sensitivity analysis was performed with the participant specified as the clustering unit. Part two modeled corridor width conditional on L_1_ > 0 by a linear mixed model with the random-effects structure described in Section 2.7.1, estimated by REML, with the overall effect of position likewise assessed by a Wald test. Both parts were fitted on the original measurement scale for consistency with L_2_ and F; a log-transformed sensitivity analysis of part two is given in Supplementary Table S1.

#### 2.7.3. Covariates and Sex

Age, sex, and BMI were added as fixed-effect covariates and their contributions assessed by likelihood ratio tests. Sex was entered as a participant-level fixed effect within the same mixed-model framework, since it does not vary within a participant; the effective sample size for these comparisons is 27 participants rather than 51 limbs. Corridor absence by sex was modeled by the same GEE logistic regression described in Section 2.7.2, with sex entered as an additional participant-level fixed effect.

#### 2.7.4. Model Diagnostics

Diagnostics comprised inspection of residual distributions (Shapiro–Wilk test, skewness, kurtosis), assessment of homoscedasticity by the Spearman correlation between absolute residuals and fitted values and by Levene’s test of residual variance across positions, identification of influential observations from standardized residuals, and sensitivity analyses using log-transformed outcomes and with influential observations excluded. Normality was assessed on the model residuals rather than on the raw values at each position, since the distributional assumption of a linear mixed model applies to the residuals.

#### 2.7.5. Multiplicity, Effect Sizes, and Sample Size

Confirmatory analyses were the effects of position on L_2_, F, and L_1_. The three outcomes were treated as separate families rather than as a single family of comparisons, since they address distinct anatomical questions. For L_2_ and F, all 15 pairwise comparisons among the six positions were performed and Bonferroni-adjusted; the adjusted comparisons are reported separately from the model coefficients, whose *p* values are unadjusted values for the individual contrast against neutral. For L_1_, pairwise comparisons among all six positions were not performed; only the five comparisons of each position against neutral were examined, and both the unadjusted p value for each contrast and the Bonferroni-adjusted value for that family of five are reported. All sex-stratified analyses, and the analyses of age and BMI, are exploratory, were not adjusted for multiplicity, and are not used to support any conclusion. Effect sizes are reported as fixed-effect coefficients with 95% confidence intervals and as within-limb standardized mean differences (Cohen’s dz) with 95% confidence intervals. For each position, the difference from the neutral position was computed within each limb, giving 51 paired differences; dz is the mean of these differences divided by their standard deviation. Its standard error was obtained from the large-sample expression SE(dz) = √(1/*n* + dz^2^/2*n*) with *n* = 51, and the confidence interval as dz ± 1.96 × SE(dz). This expression treats the 51 limbs as 51 independent units. Because 24 of the 27 participants contributed both limbs, the limbs are not fully independent, and the dz intervals therefore do not account for participant-level dependence; they should be read as descriptive summaries of the within-limb effect magnitude. Inference on position effects rests on the fixed-effect coefficients and their confidence intervals, which are estimated from the mixed models specified in Section 2.7.1 and do account for the nesting of limbs within participants. No a priori sample size calculation was performed: at the time of design no published data were available on the positional variability of these dimensions from which an expected effect size could be derived, and the target of 51 limbs was determined by recruitment within the approved study period. Post hoc power was not computed, since retrospective power is a deterministic function of the observed effect size and *p* value and conveys no information beyond the confidence intervals reported.

## 3. Results

### 3.1. Participant Characteristics

Overall, 51 upper limbs from 27 healthy adult volunteers were enrolled; 24 participants provided bilateral limb measurements. Baseline demographic and anthropometric characteristics are summarized in Table 1.

### 3.2. Approach B Safety Corridor (L_2_)

Wrist and forearm position had a significant overall effect on L_2_ (linear mixed model, *p* < 0.001). Relative to neutral, L_2_ increased in all three supination conditions and with wrist flexion alone, whereas wrist extension alone did not. Descriptive values are given in Table 2 and model estimates with within-limb effect sizes in Table 3.

In pairwise comparisons adjusted for the 15 contrasts, each supination condition exceeded both the neutral and the extension-alone position (each adjusted *p* < 0.001). The three supination conditions did not differ from one another (SUP versus E+SUP, adjusted *p* = 1.00; SUP versus F+SUP, adjusted *p* = 1.00; E+SUP versus F+SUP, adjusted *p* = 0.50).

**Table 2:** Approach B corridor (L_2_) and FPL exposure height (F) across six standardized wrist and forearm positions (*n* = 51 limbs).

Position	L_2_ (mm)	F (mm)
Neutral (NS)	5.82 ± 2.10 ^b,c,e,f^	3.24 ± 1.08 ^b,c,e,f^
Supination (SUP)	8.18 ± 2.99 ^a,c,d^	5.30 ± 1.54 ^a,c,d^
Flexion (F*)	7.20 ± 3.18 ^a,b,d,e^	4.10 ± 1.78 ^a,b,e^
Extension (E)	6.07 ± 2.27 ^b,c,e,f^	3.53 ± 1.52 ^b,e,f^
Extension + supination (E+SUP)	8.48 ± 2.61 ^a,c,d^	5.56 ± 1.54 ^a,c,d,f^
Flexion + supination (F+SUP)	7.87 ± 2.91 ^a,d^	4.73 ± 1.66 ^a,d,e^

Values are mean ± standard deviation. Superscript letters denote positions from which the value differs significantly (*p* < 0.05 after Bonferroni correction for the 15 pairwise comparisons, based on linear mixed-model contrasts): ^a^ = NS, ^b^ = SUP, ^c^ = F*, ^d^ = E, ^e^ = E+SUP, ^f^ = F+SUP. F* denotes wrist flexion, distinguished from F, the FPL exposure height. L_1_ (Approach A corridor) is presented separately in Table 4, because the corridor was absent in a variable proportion of limbs and therefore required a two-part analysis. Abbreviations: E, wrist extension; E+SUP, wrist extension with forearm supination; F, FPL exposure height; F*, wrist flexion; F+SUP, wrist flexion with forearm supination; FPL, flexor pollicis longus; L_2_, Approach B corridor; NS, neutral; SUP, forearm supination.

**Table 3:** Linear mixed-model estimates and within-limb effect sizes for the effect of wrist and forearm position on L_2_ and F.

Position (vs. NS)	L_2_: β (95% CI), mm	*p*	L_2_: dz (95% CI)	F: β (95% CI), mm	*p*	F: dz (95% CI)
Supination (SUP)	+2.36 (1.79, 2.93)	<0.001	1.28 (0.91, 1.65)	+2.07 (1.64, 2.50)	<0.001	1.35 (0.97, 1.73)
Flexion (F*)	+1.38 (0.82, 1.95)	<0.001	0.63 (0.33, 0.93)	+0.87 (0.44, 1.30)	<0.001	0.50 (0.20, 0.79)
Extension (E)	+0.26 (−0.31, 0.82)	0.375	0.13 (−0.14, 0.41)	+0.29 (−0.14, 0.72)	0.186	0.20 (−0.08, 0.47)
Extension + supination (E+SUP)	+2.66 (2.10, 3.23)	<0.001	1.23 (0.87, 1.60)	+2.32 (1.89, 2.75)	<0.001	1.55 (1.14, 1.96)
Flexion + supination (F+SUP)	+2.05 (1.48, 2.61)	<0.001	0.89 (0.57, 1.22)	+1.49 (1.07, 1.92)	<0.001	0.83 (0.51, 1.14)

β represents the average change in each outcome (mm) for a given position relative to neutral, as estimated from a linear mixed model; for any position, the model-estimated mean equals the neutral estimate plus β. The model-estimated marginal means at the neutral position were 5.83 mm for L_2_ and 3.25 mm for F; because these are model-based values, they differ slightly from the raw means in Table 2. dz is the within-limb standardized mean difference (Cohen’s dz), calculated as the mean of the paired within-limb differences from the neutral position divided by their standard deviation; values of approximately 0.2, 0.5, and 0.8 correspond to small, medium, and large effects, respectively. *p* values in this table are unadjusted values for the individual contrast against the neutral position; the Bonferroni-adjusted comparisons among all six positions for the same outcomes are denoted by superscripts in Table 2. Confidence intervals for dz were computed from the large-sample standard error √(1/*n* + dz^2^/2*n*) with *n* = 51 limbs and treat limbs as independent; they do not account for the dependence between the two limbs of the same participant, unlike the confidence intervals for β. See Section 2.7.5. The models included wrist and forearm position as a fixed effect (neutral as the reference) and random intercepts for participant and for limb nested within participant, with a compound-symmetric covariance structure at each level. Variance components were, for L_2_, 2.399 (participant), 2.786 (limb within participant), and 2.123 (residual); and for F, 0.727, 0.432, and 1.222, respectively. The corresponding intraclass correlation coefficients were 0.328 at the participant level and 0.710 at the level of limb within participant for L_2_, and 0.305 and 0.487 for F. Final parameter estimates were obtained by restricted maximum likelihood; models differing in fixed effects were re-estimated by full maximum likelihood for likelihood ratio testing. Adding age, sex, and body mass index as covariates left the estimates essentially unchanged, and none was significant. F* denotes wrist flexion, distinguished from F, the FPL exposure height; the asterisk is part of the abbreviation and is not a footnote marker. Abbreviations: β, fixed-effect coefficient; CI, confidence interval; dz, within-limb standardized mean difference; E, wrist extension; E+SUP, wrist extension with forearm supination; F, FPL exposure height; F*, wrist flexion; F+SUP, wrist flexion with forearm supination; FPL, flexor pollicis longus; L_2_, Approach B corridor; NS, neutral; SUP, forearm supination.

### 3.3. FPL Exposure Height (F)

Position also had a significant overall effect on F (*p* < 0.001). Relative to neutral, F increased in all conditions except wrist extension alone and was numerically greatest in E+SUP (Table 2 and Table 3).

In adjusted pairwise comparisons, F in E+SUP exceeded that in the neutral, flexion-alone, and extension-alone positions and that in F+SUP, but did not differ from supination alone (Table 2). The model estimate was highest for E+SUP, but the difference from supination alone was not significant (Table 3).

Model diagnostics were acceptable for both outcomes. Residual skewness and kurtosis were small (L_2_, 0.15 and 0.93; F, 0.16 and 0.38), although the Shapiro–Wilk test reached significance at this sample size (L_2_, W = 0.988, *p* = 0.013; F, W = 0.991, *p* = 0.044). Residual variance was homogeneous across positions (Levene’s test: L_2_, *p* = 0.953; F, *p* = 0.163). Log-transformed sensitivity analyses gave the same pattern of results (for example, L_2_ in E+SUP, +49.4%; F, +76.5%; both *p* < 0.001), as did analyses excluding the three observations with standardized residuals exceeding 3 in absolute value (all positional coefficients changed by 0.08 mm or less).

### 3.4. Approach A Safety Corridor (L_1_)

The Approach B corridor was measurable in every limb at every position, whereas the Approach A corridor was absent (L_1_ = 0) in 12–28% of limbs depending on position (Table 4).

In the first part of the hurdle model, corridor absence was more frequent than in neutral in the extension and extension-with-supination positions, but no contrast remained significant after Bonferroni correction and the overall effect of position was not significant (Table 4a). In the second part, among limbs in which the corridor was present, its width differed significantly across positions and was narrower than neutral in both supination conditions (Table 4b). In a sensitivity analysis specifying the participant rather than the limb as the clustering unit, the coefficients were essentially unchanged and the overall effect of position was of borderline significance (Wald χ^2^(5) = 11.19, *p* = 0.048).

The two parts of the model are concordant: the position in which the corridor was most often absent was also the position in which, where present, it was narrowest.

**Table 4:** Two-part (hurdle) analysis of the Approach A corridor (L_1_) across six wrist and forearm positions. (**a**) Probability that the corridor was absent (L_1_ = 0). Generalized estimating equations (logistic); 306 observations from 51 limbs in 27 participants. (**b**) Corridor width conditional on L_1_ > 0. Linear mixed model; 246 observations from 50 limbs in 27 participants; one limb had no measurable corridor in any position and therefore did not contribute.

(**a**)						
**Position**	**Limbs with L_1_ = 0, *n* (%)**		**Log-Odds vs. NS**	**95% CI**	***p***	***p***_**adj**_
Neutral (NS), reference	6 (11.8)		—	—	—	—
Supination (SUP)	9 (17.6)		+0.47	−0.34, 1.29	0.255	1.000
Flexion (F*)	9 (17.6)		+0.47	−0.45, 1.40	0.317	1.000
Extension (E)	12 (23.5)		+0.84	+0.19, 1.48	0.011	0.057
Extension + supination (E+SUP)	14 (27.5)		+1.04	+0.17, 1.92	0.020	0.098
Flexion + supination (F+SUP)	10 (19.6)		+0.60	−0.23, 1.44	0.155	0.776
(**b**)						
**Position**	***n***	**Mean ± SD (mm)**	**β vs. NS (mm)**	**95% CI**	***p***	***p*****_adj_**
Neutral (NS), reference	45	6.53 ± 5.24	—	—	—	—
Supination (SUP)	42	4.96 ± 3.80	−1.82	−2.76, −0.89	<0.001	<0.001
Flexion (F*)	42	5.68 ± 5.09	−1.13	−2.07, −0.19	0.019	0.094
Extension (E)	39	6.05 ± 4.16	−1.02	−1.98, −0.07	0.036	0.179
Extension + supination (E+SUP)	37	5.24 ± 3.98	−1.93	−2.90, −0.95	<0.001	<0.001
Flexion + supination (F+SUP)	41	6.54 ± 5.75	−0.36	−1.30, +0.59	0.458	1.000

For (**a**), the overall effect of position was Wald χ^2^(5) = 9.64, *p* = 0.086; for (**b**), Wald χ^2^(5) = 24.76, *p* < 0.001. Panel (**a**) was estimated by generalized estimating equations with the limb as the clustering unit, an exchangeable working correlation structure, and robust standard errors; its coefficients are population-averaged log-odds of corridor absence relative to the neutral position, and the overall effect of position is a Wald test. Panel (**b**) is a linear mixed model including random intercepts for participant and for limb nested within participant, estimated by restricted maximum likelihood; the overall effect of position is likewise a Wald test. Panel (**b**) coefficients are differences in corridor width in mm relative to the neutral position, estimated on the original measurement scale; a log-transformed sensitivity analysis is presented in Supplementary Table S1. In that sensitivity analysis the effects for SUP and E+SUP were unchanged, whereas the effect for E did not reach significance; the primary conclusions rest on the supination conditions. The number of limbs contributing to (**b**) differs across positions because the corridor was absent in a variable proportion of limbs. *p* is the unadjusted *p* value for the contrast against the neutral position; *p*_adj_ is the Bonferroni-adjusted value for the family of five comparisons against neutral. L_1_ was recorded as zero when both structures were identifiable but no usable corridor was present between them, either because they were in direct apposition or because their mediolateral order was reversed; a zero value denotes the operational absence of a usable corridor rather than a literal 0 mm separation. F* denotes wrist flexion; the asterisk distinguishes it from F, the flexor pollicis longus exposure height reported in Table 2 and Table 3. Abbreviations: CI, confidence interval; E, wrist extension; E+SUP, wrist extension with forearm supination; F*, wrist flexion; F+SUP, wrist flexion with forearm supination; L_1_, Approach A corridor; NS, neutral; SD, standard deviation; SUP, forearm supination.

### 3.5. Reliability of Measurements

Intra-rater reliability was excellent for both raters across all measurements (ICC(A,1), 0.93–1.00). Inter-rater reliability, based on the average-measures model corresponding to the two-rater mean, was good to excellent across the six wrist and forearm positions (ICC(A,2), 0.76–0.95), with pooled estimates of 0.88 for L_1_, 0.93 for L_2_, and 0.90 for F; the corresponding single-measures estimates ranged from 0.61 to 0.91 (pooled 0.78, 0.86, and 0.82, respectively). These values reflect measurement repeatability on stored images and not the reproducibility of ultrasound acquisition, since all images were acquired by a single sonographer and both raters measured the same images.

### 3.6. Exploratory Analyses of Age, Sex, and Body Mass Index

The following analyses were exploratory and were not adjusted for multiplicity. Including age, sex, and BMI as covariates did not change the positional effects. None was independently associated with L_2_ (likelihood ratio *p* = 0.707, 0.322, and 0.237) or with F (*p* = 0.628, 0.279, and 0.110), and their joint contribution was non-significant for both (L_2_, χ^2^(3) = 1.81, *p* = 0.614; F, χ^2^(3) = 2.90, *p* = 0.408). Sex was not independently associated with any outcome when modeled as a participant-level fixed effect (L_2_, β = +0.75 mm, *p* = 0.34; F, β = −0.42 mm, *p* = 0.29; corridor absence, log-odds +1.02, *p* = 0.08; corridor width where present, β = −2.39 mm, *p* = 0.11). Sex-stratified descriptive values and the corresponding mixed-model estimates are provided in Table S2.

## 4. Discussion

Ultrasound guidance has reduced but not eliminated the risk of neurovascular injury during FPL injection, and existing work has addressed this by refining needle technique while treating limb position as a fixed feature of the procedural set-up. To our knowledge this is the first study to ask whether limb position itself can be used to alter the sonographic anatomy in a favorable direction, and it does so with a maneuver that requires no additional equipment and can be applied before the needle is introduced. The practical implication is concrete: supinating the forearm before scanning begins leaves the operator working within a wider corridor than the pronated posture in which the spastic limb usually presents.

This prospective study shows that wrist and forearm positioning alters the sonographic anatomy relevant to ultrasound-guided FPL injection, and yields three principal findings. First, the Approach B corridor (L_2_) was wider in all supination postures than in neutral, and the three supination postures did not differ from one another. Second, F increased under the same conditions, with the highest values in E+SUP, which nonetheless did not differ significantly from supination alone. Third, unlike L_2_ and F, the Approach A corridor (L_1_) was frequently unmeasurable and, where measurable, was narrowed rather than widened by supination, so that the posture favoring Approach B is unfavorable for Approach A. Taken together, forearm supination combined with Approach B produced the largest sonographic dimensions among the positions investigated.

These results both build on and depart from previous work. As in earlier studies, our study used ultrasound to identify a safe route to the FPL in the distal forearm. Nonetheless, those investigations were designed to guide perpendicular needle insertion for diagnostic EMG and characterized the relevant anatomy in a single, static neutral posture, typically relying on fixed surface landmarks or arterial pulse palpation to define a safe entry point [20,21,22]. No study examined how the access corridor changes as the wrist and forearm move. Because the FPL and its overlying neurovascular structures shift with forearm rotation, a description obtained in one fixed posture cannot identify the position that increases the measured corridor. This study advances that earlier work in two respects. It addresses therapeutic, in-plane injection rather than diagnostic needling, and it demonstrates that a simple, reproducible maneuver (forearm supination) significantly widens the injection corridor, enlarging L_2_ by approximately 40% (about 2.4 mm) relative to neutral. It therefore identifies patient positioning as a modifiable variable that merits deliberate consideration during procedural set-up, rather than an incidental part of it.

One anatomical hypothesis that would account for the widening of L_2_ relates to the geometry of forearm rotation. During supination the radius rotates about the ulna, and it is plausible that the apex of the radial cortex thereby comes to lie farther from the CV in the transverse plane, enlarging the bone-to-vein corridor [31,32]. We did not, however, measure the displacement of the individual structures, and our data cannot distinguish between the possible contributions to the observed change: displacement of the radial cortex, movement of the CV, deformation of the intervening soft tissues, or some combination of these. The pattern of our results is at least compatible with a rotation-driven mechanism, in that the increase in L_2_ was present in all three supination conditions and was of similar magnitude irrespective of concomitant wrist flexion or extension, which suggests that forearm rotation rather than the accompanying wrist angle is the component of the maneuver most closely associated with L_2_ enlargement. This remains an anatomical hypothesis that requires direct verification, for example, by tracking the individual structures across postures.

The role of wrist extension was less clear-cut. F reached its highest values when extension was combined with supination, and the corresponding model estimate exceeded that of supination alone (+2.32 versus +2.07 mm); the two conditions did not, however, differ statistically, and the same was true for L_2_. On these data, wrist extension cannot be said to add to what supination already achieves. A cadaveric study has shown that wrist extension increases tension within the FPL tendon [33], which might draw the muscle belly into a more superficial relationship with the radial cortex; our findings are compatible with such an effect but do not demonstrate it. In practice the choice between supination alone and supination with wrist extension may therefore be made on other grounds—patient comfort, available range of motion, and the ease of holding the position during the procedure—since neither posture has been shown to be superior for the dimensions measured here.

Whether a difference of 2 to 3 mm matters clinically cannot be settled by anatomical measurement alone, but its scale can be stated. The corridor available in the neutral position averaged 5.8 mm, so an increase of 2.4 mm represents roughly a 40% gain within a space that is itself less than 1 cm across. A needle of the caliber used for chemodenervation has an outer diameter of approximately 0.5 mm; the observed increase therefore corresponds to four to six needle widths of additional clearance between the structures that must be avoided. Against this, the absolute distances remain small, and because no injections were performed we cannot claim that the wider corridor translates into fewer punctures of the vein or nerve, better needle visualization, or improved clinical outcomes. The increase is not, however, self-evidently negligible when set against the dimensions of the instrument being passed through the space. The angle at which that instrument must be advanced is a separate consideration, addressed below.

In contrast, L_1_ was narrowed by supination and was often unmeasurable, because the CV and the SBRN were in direct apposition. This pattern corroborates the well-documented interindividual variation in the distal forearm course of these two structures [17,18,34], and it helps explain why positioning could not reliably improve Approach A. A zero value for L_1_ should therefore be interpreted as the operational absence of a usable corridor, rather than a literal 0 mm distance. Thus, these observations favor Approach B over Approach A as the dependable route because it yielded a measurable corridor in every limb and at every position tested.

It should be noted that the two approaches differ not only in corridor width but in the needle angle they require (Figure 2). Approach A is directed more steeply, since the needle descends between the CV and the SBRN over a short horizontal distance, whereas Approach B is entered at a shallower angle in order to pass beneath the CV and above the radial cortex. The trade-offs differ accordingly: a steeper trajectory reaches the muscle over a shorter intramuscular path but returns less of the ultrasound beam to the transducer, so the needle is harder to keep in view; a shallower trajectory favors needle visualization but lengthens the path within the muscle. Whether the wider corridor observed in supination alters this balance is not established by our data.

The value of the maneuver may also depend on operator experience. An experienced sonographer may be able to complete the injection reliably from the neutral position, compensating for a narrow corridor with fine adjustments of needle angle and transducer pressure. A less experienced operator has less margin for such compensation, and a wider corridor may translate more directly into a more forgiving procedure. Conversely, Approach B requires the needle to be advanced through a narrow window between the CV and the bony surface over a longer intramuscular path, which is itself technically demanding, and whether the net benefit is greater for less experienced operators cannot be determined from anatomical measurements alone. This would be a useful question for a study linking positioning to procedural performance.

Several features increase confidence that these positional effects are genuine. The effect was internally consistent: it appeared across all supination conditions and remained essentially unchanged after adjustment for age, sex, and BMI, which argues against demographic confounding. The finding is also biomechanically plausible, in that it is consistent with the established kinematics of forearm rotation rather than appearing as an isolated or unexplained association. Moreover, the underlying principle that deliberate positioning can shift vascular structures relative to the intended needle path is already exploited in other ultrasound-guided procedures, such as stellate ganglion block [25]. From a practical standpoint, the proposed maneuver requires no additional equipment and integrates readily into existing real-time ultrasound workflows [11,12]. We also examined whether the fixed acquisition order could have produced a systematic drift; residuals of the mixed-effects models showed no monotonic trend across the sequence (L_2_, Spearman rho = −0.003, *p* = 0.96; F, rho = −0.03, *p* = 0.66). Taken together, these findings indicate that the proposed posture increases the sonographic dimensions measured under the specific experimental conditions evaluated here. Whether this translates into improved injection accuracy, reduced neurovascular injury, better needle visualization, or greater clinical effectiveness was not assessed.

This study has several limitations. First, it was performed in healthy adults with a full range of motion, whereas FPL chemodenervation is indicated in spasticity, where contracture, altered muscle architecture, increased passive stiffness, and chronic vascular adaptation may modify the spatial relationships among the FPL, the SBRN, the CV, and the radial cortex, and may restrict the recommended posture altogether. The cohort was also elderly (mean age 75 years), a consequence of the recruitment setting rather than a design choice; no age stratum was targeted. Reduced muscle bulk, intramuscular fat, and diminished venous tone accompany ageing and could affect the measurements—F in particular. The findings should therefore be regarded as preliminary anatomical evidence requiring confirmation in the target population [7,35].

Second, what was measured constrains what can be concluded. L_1_ and L_2_ were obtained on a single transverse plane, and a width in that plane does not guarantee a three-dimensional corridor free of neurovascular structures along the whole trajectory, nor does it establish technical feasibility, which also depends on needle angulation, tissue depth, path length, and continuous visualization of the tip. Both should be read as indirect surrogates of accessibility rather than validated measures of procedural safety. All measurements were also obtained at one longitudinal level, chosen as the level of maximal FPL exposure in neutral. Holding the level fixed isolates the effect of posture but leaves the optimal injection level undetermined: what is reported is the effect of posture at one level, not the effect of level.

Third, the measurements may partly reflect factors other than the underlying anatomy. The apex of the radial cortex is image-dependent; transducer tilt and pressure were not recorded, and forearm rotation turns the radius itself, so the highest visible cortical margin may correspond to a different part of the bony surface in different positions. We regard this as intrinsic to the measurement, since the structure obstructing the needle in a given posture is the surface that is in fact most prominent in that posture, but L_2_ should be read as a posture-specific sonographic dimension rather than the displacement of a fixed point. The CV raises a comparable problem: it is compliant, and although limb position was held constant, room temperature was not regulated and hydration and preceding activity were not recorded. Because both corridors are measured from the venous wall, variation in venous filling cannot be separated from the positional effect.

Fourth, the posture is not defined by a standardized joint angle: supination and wrist extension were taken to each participant’s maximal available range, and joint angles were not measured. This corresponds to practice, but maximal range varies between individuals, so the reproducibility of the posture is limited, a constraint likely to be greater in patients. The six positions were also acquired in fixed rather than randomized order, which may introduce systematic effects from repeated compression, venous redistribution, or progressive soft-tissue relaxation. Returning the limb to neutral before each posture and supporting it passively throughout limits this risk, and the residual analysis argues against cumulative drift, but an effect acting uniformly across all six acquisitions cannot be detected, since neutral was not re-imaged at the end. Order-related bias therefore cannot be excluded.

Fifth, the reported ICCs reflect measurement rather than scan–rescan reliability, since a single sonographer acquired all images and both raters measured the same images; the positional differences rest on the assumption that the imaging plane was reproduced consistently. The examiner was aware of both the position and the hypothesis, and expectation could have influenced acquisition; the skin mark fixed the scanning level beforehand and measurement was independent and off-line, but acquisition itself remained unblinded. These conditions also differ from an actual procedure, in which structures are identified in real time rather than tracked before storage and read off-line, and recognition of small neural structures depends on experience and on dynamic rather than static assessment [27]; the dimensions reported here may therefore overstate what is reliably identifiable during injection. Finally, no a priori sample size calculation was performed, and the study was not powered for participant-level characteristics, for which the effective sample size is 27 rather than 51. These limitations are best addressed by adequately powered, multicenter studies in patients with spasticity that link positioning to injection outcomes, incorporate multiple operators and blinded, randomized-order measurements, and sample more diverse populations and forearm levels.

In conclusion, in healthy forearms, forearm supination significantly increased the sonographic safety corridor and target exposure for ultrasound-guided FPL injection via Approach B, whereas positioning did not increase the corridor for Approach A. The three supination postures did not differ from one another for the corridor, and adding wrist extension to supination did not significantly increase target exposure beyond supination alone; the choice among them may therefore rest on patient comfort and available range of motion. These findings are consistent with, though they do not establish, a rotation-based mechanism, and they should be validated in patients with spasticity before routine clinical adoption.

## Figures and Tables

**Table 1:** Baseline demographic and anthropometric characteristics of study participants.

Characteristic	Value
Total participants, *n*	27
Sex, *n* (%)	
Male	15 (55.6)
Female	12 (44.4)
Total limbs, *n*	51
Sex, *n* (%)	
Male	29 (56.9)
Female	22 (43.1)
Side evaluated, *n* (%)	
Left	24 (47.1)
Right	27 (52.9)
Age (years)	75.1 ± 5.4
Height (cm)	159.4 ± 8.7
Weight (kg)	62.9 ± 7.7
BMI (kg/m^2^)	24.8 ± 2.5

Continuous variables are expressed as mean ± SD and were calculated at the participant level (*n* = 27); limb counts and side distribution refer to the 51 limbs analyzed. Categorical variables are *n* (%). BMI, body mass index; SD, standard deviation.

## Data Availability

The original contributions presented in this study are included in the article and Supplementary Materials. Further inquiries can be directed to the corresponding author.

## Supplementary Materials

The following supporting information can be downloaded at: https://www.mdpi.com/article/10.3390/diagnostics16162674/s1, Table S1: Log-transformed sensitivity analysis of the Approach A corridor width (panel (b) of Table 4); Table S2: Exploratory analysis of sex.

## Abbreviations

The following abbreviations are used in this manuscript:

BMI	body mass index
BoNT-A	botulinum toxin type A
CI	confidence interval
CV	cephalic vein
dz	within-limb standardized mean difference (Cohen’s dz)
E	wrist extension
E+SUP	wrist extension with forearm supination
EMG	electromyography
F	FPL exposure height (sonographic height of muscle visible above the radial cortex)
F*	wrist flexion
F+SUP	wrist flexion with forearm supination
FPL	flexor pollicis longus
GEE	generalized estimating equations
ICC	intraclass correlation coefficient
IRB	Institutional Review Board
NS	neutral
RA	radial artery
REML	restricted maximum likelihood
SBRN	superficial branch of the radial nerve
SD	standard deviation
SUP	forearm supination
