# Supplementary material for: Forearm Supination Increases the Safety Corridor and Target Exposure for Ultrasound-Guided Flexor Pollicis Longus Injection: A Prospective Study in Healthy Adults
Source: Diagnostics (Basel). 2026 Aug 21;16(16):2674. doi: 10.3390/diagnostics16162674 (PMC13512723; doi:10.3390/diagnostics16162674)
Supplement: Supplementary file 1 [file diagnostics-16-02674-s001.zip › diagnostics-4486095-supplementary.pdf]

Supplementary Materials

Forearm Supination Increases the Safety Corridor and Target Exposure for Ultrasound-Guided Flexor Pollicis Longus Injection: A Prospective Study in Healthy Adults

Table S1. Log-transformed sensitivity analysis of the Approach A corridor width (panel (b) of Table 4).

**Position (vs NS)**	**Change in corridor width (%)**	**95% CI (%)**	***p***
Supination (SUP)	−24.0	−39.1 to −5.3	0.015
Flexion (F*)	−20.0	−35.98 to −0.01	0.050
Extension (E)	−9.9	−28.1 to +12.8	0.362
Extension + supination (E+SUP)	−30.6	−44.9 to −12.5	0.002
Flexion + supination (F+SUP)	−14.7	−31.8 to +6.6	0.162

The model was identical to panel (b) of Table 4 of the main text, except that the corridor width was log-transformed before fitting; coefficients are therefore back-transformed and expressed as the percentage change in corridor width relative to the neutral position. The analysis was restricted to observations in which the corridor was present ($L_1 > 0$): 246 observations from 50 limbs in 27 participants. The model included wrist and forearm position as a fixed effect and random intercepts for participant and for limb nested within participant. *p* values in this table are unadjusted. The effects for SUP and E+SUP remained significant, whereas the effect for E did not; the conclusions of the main analysis rest on the supination conditions. F* denotes wrist flexion; the asterisk distinguishes it from F, the flexor pollicis longus exposure height reported in the main text. Abbreviations: CI, confidence interval; E, wrist extension; E+SUP, wrist extension with forearm supination; F*, wrist flexion; F+SUP, wrist flexion with forearm supination; L_1, Approach A corridor; NS, neutral; SUP, forearm supination.

Table S2. Exploratory analysis of sex.

Part 1. Descriptive values by sex (mean ± SD, mm).

Position	L_2, men (n = 29 limbs)	L_2, women (n = 22 limbs)	F, men (n = 29 limbs)	F, women (n = 22 limbs)
Neutral (NS)	6.09 ± 2.08	5.47 ± 2.12	3.14 ± 1.04	3.36 ± 1.16
Supination (SUP)	8.29 ± 3.21	8.03 ± 2.73	5.09 ± 1.70	5.58 ± 1.29
Flexion (F*)	7.91 ± 3.33	6.27 ± 2.78	4.08 ± 1.75	4.14 ± 1.86
Extension (E)	6.45 ± 2.56	5.59 ± 1.78	3.35 ± 1.61	3.75 ± 1.40
Extension + supination (E+SUP)	8.51 ± 2.89	8.45 ± 2.25	5.19 ± 1.48	6.04 ± 1.50
Flexion + supination (F+SUP)	8.48 ± 3.23	7.06 ± 2.25	4.62 ± 1.83	4.88 ± 1.42

Part 2. Sex as a participant-level fixed effect (men versus women).

Outcome	Estimate	95% CI	*p*
Approach B corridor (L_2), mm	+0.75	−0.79 to +2.29	0.34
FPL exposure height (F), mm	−0.42	−1.21 to +0.36	0.29
Corridor absence (L_1 = 0), log-odds	+1.02	−0.13 to +2.18	0.08
Approach A corridor width (L_1 > 0), mm	−2.39	−5.35 to +0.58	0.11

Part 1 gives raw descriptive values. In Part 2, sex was entered as a participant-level fixed effect within the same mixed-model framework used in the main analysis, since sex does not vary within a participant; the effective sample size for these comparisons is therefore 27 participants (15 men, 12 women) rather than 51 limbs. Estimates for L_2, F, and the Approach A corridor width are differences in mm for men relative to women; the estimate for corridor absence is a population-averaged log-odds ratio obtained from the logistic regression estimated by generalized estimating equations described in Section 2.7.2 of the main text. All analyses in this table are exploratory, were not adjusted for multiplicity, and are not used to support any conclusion. F* denotes wrist flexion; the asterisk distinguishes it from F, the flexor pollicis longus exposure height. Abbreviations: CI, confidence interval; E, wrist extension; E+SUP, wrist extension with forearm supination; F, flexor pollicis longus exposure height; F*, wrist flexion; F+SUP, wrist flexion with forearm supination; L_1, Approach A corridor; L_2, Approach B corridor; NS, neutral; SD, standard deviation; SUP, forearm supination.
